# Clinicopathological features of two cases of *ETV6-NTRK3* rearranged papillary thyroid carcinoma: a case report

**DOI:** 10.3389/fonc.2024.1332522

**Published:** 2024-05-28

**Authors:** Jing Ke, Minghua Cao, Wenzhong Zhang, Hua Huang, Ping Chen, Jinhua Liu, Dan Shan, Jie Ke, Zerui Wang, Junchen Liu, Yuan Li, Sheng Xiao

**Affiliations:** ^1^ Department of Thyroid and Breast Surgery, Affiliated Hospital of Nantong University, Nantong, China; ^2^ Department of Thyroid and Breast Surgery, The First People’s Hospital of Jiashan County, Jianxing, China; ^3^ Department of General Surgery, Shanghai Pudong New Area People’s Hospital, Shanghai, China; ^4^ Department of Cytogenetics, Sano Suzhou Precision Medicine Co. Ltd., Suzhou, China; ^5^ Department of Pathology, Harvard Medical School, Brigham and Women’s Hospital, Boston, MA, United States

**Keywords:** clinicopathological features, thyroid carcinoma, ETV6-NTRK3 rearrangement, the follicular variant of papillary thyroid carcinoma, case report

## Abstract

Rearrangements involving the neurotrophic-tropomyosin receptor kinase (NTRK) gene family (*NTRK1, NTRK2*, and *NTRK3*) have been identified as drivers in a wide variety of human cancers. However, the association between *NTRK* rearranged thyroid carcinoma and clinicopathological characteristics has not yet been established. In our study, we retrospectively reviewed medical records of thyroid cancer patients and identified 2 cases with *NTRK* rearrangement, no additional molecular alterations were observed in either of these cases. The fusion of the rearrangement in both cases was *ETV6*(E4)::*NTRK3*(E14). By analyzing the clinicopathological features of these two cases, we found that both were characterized by multiple tumor nodules, invasive growth, and central lymph node metastases, indicating the follicular subtype of papillary thyroid carcinoma. Immunohistochemical staining profiles showed CD56-, CK19+, Galectin-3+, HBME1+. These clinicopathological features suggest the possibility of *ETV6-NTRK3* rearranged thyroid carcinoma and highlight the importance of performing gene fusion testing by FISH or NGS for these patients.

## 1 Introduction

Thyroid cancer ranks as the ninth most common cancer worldwide. Despite significant advancements in the management of thyroid cancer, there remain critical challenges in its diagnosis and treatment, leading to poor overall survival rates for certain aggressive subtypes and patients with metastatic thyroid cancer. Accurate diagnosis of thyroid cancer is crucial for guiding appropriate treatment and patient management. Over the past 15 years, the significance of molecular biology in thyroid pathology has not only revolutionized the field but also underscored the intrinsic value of classical histopathology. Pathologists have long identified patterns indicative of specific molecular changes, but the incorporation of molecular tools into their toolkit has bolstered our capacity to prognosticate and predict the effectiveness of targeted treatments. In the new fifth edition of the World Health Organization (WHO) histologic classification of thyroid neoplasms, the classification of thyroid tumors has evolved based on classic histopathology and molecular pathogenesis (1).

Papillary thyroid cancer (PTC) is the most common type of thyroid cancer in China, accounting for over 90% of all thyroid cancer cases (2). The prognosis and treatment of thyroid cancer depend on the tumor type and its stage. Early diagnosis and appropriate treatment can significantly reduce mortality rates and improve prognosis. Apart from age, tumor size, and lymph node involvement, the behavior of PTC is also influenced by the morphological subtypes of genetic mutations (3). Certain subtypes, such as the tall cell subtype (4), the columnar cell subtype (5), and the recently identified hobnail subtype are associated with more aggressive behavior and poorer overall survival (6). In recent years, there has been increasing evidence of molecular alterations that correspond to these pathological subtypes. The Cancer Genome Atlas (TCGA) conducted a study on 496 PTCs, confirming the significance of mutations in *BRAF* and *RAS* genes, as well as fusion mutations in *RET* and *NTRK1.* The study also revealed correlations between morphology and genetic alterations, with *BRAF ^V600E^*-positive tumors being associated with the conventional and hypercellular variants of PTC, while *RAS*-positive tumors were linked to the follicular variant of PTC. However, the clinicopathological association of *NTRK*-rearranged thyroid carcinoma has not yet been established (7).

Rearrangements involving the neurotrophic-tropomyosin receptor kinase (*NTRK*) gene family, namely *NTRK1, NTRK2*, and *NTRK3*, have been identified as drivers in a wide range of human cancers (8–10). These rearrangements result in NTRK gene fusions with various partner genes, which confer oncogenic potential by generating chimeric Trk proteins. These proteins constitutively activate kinase function, leading to the downstream stimulation of cellular proliferation through the RAS/RAF/MAPK pathway and PI3K-AKT signaling pathways (11, 12). Larotrectinib and entrectinib, both tyrosine kinase inhibitors (TKIs), have been proven to be safe and effective treatment options for patients with *NTRK* fusion-positive solid tumors. Studies have shown that larotrectinib and entrectinib yield significant responsiveness and minimal primary resistance in thyroid tumors (13–17). However, due to the high cost of testing, not all patients with thyroid cancer who may have an *NTRK* rearrangement can be tested and treated. In this report, we present the clinicopathological data of two patients with *ETV6-NTRK3*, which may provide valuable insights for identifying this specific type of thyroid cancer.

## 2 Materials and methods

### 2.1 Patient selection

For this study, we retrospectively reviewed medical records of thyroid cancer patients treated at our center from January 2021 to December 2022. Two patients were identified from the hospital pathology database. Inclusion criteria were: histologically confirmed thyroid cancer, availability of formalin-fixed paraffin-embedded (FFPE) tissue blocks, complete clinical and pathological data, the molecular pathologic test results were negative for *BRAF* mutation. Patients with a history of other malignancies, inadequate tissue specimens were excluded. This study was approved by the institutional ethics committee.

### 2.2 Histology and immunohistochemistry

Hematoxylin and eosin (HE) staining and immunohistochemical (IHC) staining were performed on 4 μm thick formalin-fixed paraffin-embedded (FFPE) sections from the two patients with NTRK-rearranged thyroid cancer. The primary antibodies used were CD56, Cytokeratin 19 (CK19), Galectin-3 (Thermo Fisher Scientific, Waltham, MA), HBME1 (Dako; Carpinteria, CA), and Pan-TRK (pre-diluted; Ventana, Oro Valley, AZ). Appropriate positive and negative controls were included.

### 2.3 Fluorescence *in situ* hybridization

FISH analysis was conducted on FFPE sections using commercially available break-apart probe sets (NTRK3) from Guangzhou LBP Medicine Science & Technology Co., Ltd. (Guangzhou, PRC.). Fifty interphase nuclei were analyzed. The FFPE specimen underwent standard FISH pretreatment, hybridization, and fluorescence microscopy following specimen-specific protocols. The FISH analysis was independently evaluated by two qualified clinical cytogenetic technologists and interpreted by a board-certified (ABMGG) clinical cytogeneticist.

### 2.4 Targeted RNA next-generation sequencing

Total RNA was isolated from. the representative 10-μm FFPE tumor tissue sections using the FFPE Total RNA Miniprep System (Cat: Z1002, Promega, USA). Reverse transcription (ABclona, Cat #RK20353 and RK20346, China), end repairing (Enzymatics, Cat #Y9140-LC-L, USA), dA-tailing (ThermoFisher, Waltham, MA), and adaptor ligation (Enzymatics, Cat #L6030-LC-L, USA) were performed according to standard NGS protocols. PCR enrichment was conducted using primers specific to a group of 73 genes commonly associated with thyroid malignancies. The PCR products were then sequenced using a NovaSeq sequencer (Illumina, USA). The sequencing results were analyzed using SeqNext software (JSI, Ettenheim, Germany).

## 3 Results

### 3.1 Clinical and staging parameters

Clinical and staging parameters are summarized in **Table 1**. The two patients were 32-year-old and 57-year-old women, both were euthyroid, none of the patients with available information had a family history of thyroid cancer or personal history of prior irradiation. Fine needle aspirate biopsy (FNAB) findings were not available in them. both patients underwent total thyroidectomy and a formal central compartment dissection.

**Table 1:** Clinical and staging parameters for ETV6-NTRK3 translocated papillary thyroid carcinoma.

Case	1	2
Age	32Y	57Y
Sex	F	F
Type of Surgery	TT, CCND	TT, CCND
Functional Status	Euthyroid	Euthyroid
Family History of Thyroid Cancer	No	No
Prior History of Irradiation	No	No
FNAB	No	No
pTNM	pT1bN1aM0	pT3aN1aM0
Post-operative I131	No	No
Progression Free Survival (months)	25M	23M

TT, total thyroidectomy; CCND, central compartment neck dissection.

The tumor size of the two cases was 1.5cm and 1.2 cm respectively, both had separate foci of papillary thyroid carcinoma and without extrathyroidal extension. Nodal metastases were noted in both patients with lymph node sampling (1/5 and 5/5 respectively); None of the patients received radioactive iodine. Both patients were free of disease at time of last follow-up (25 and 23months), satisfied with the treatment results, in good mental condition, and showed no signs of anxiety or depression.

### 3.2 Histological, immunophenotypic, and molecular characterization

**Figure 1:**
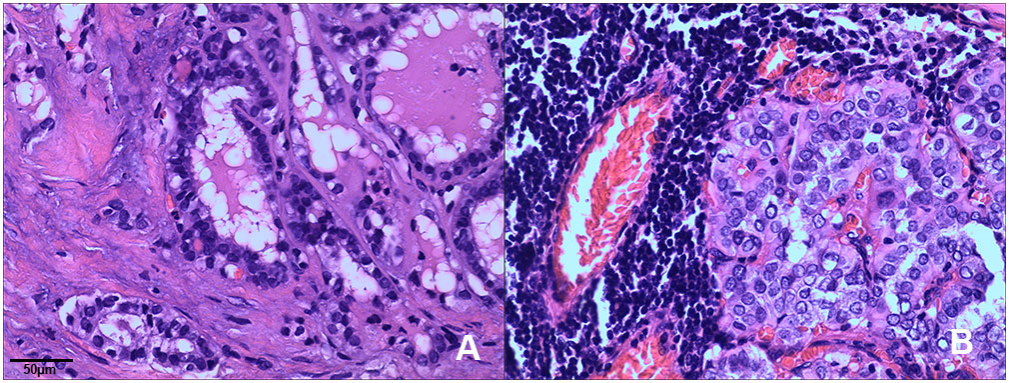
Histopathological features of *ETV6-NTRK3* rearranged thyroid cancer. Tumors typically exhibited a combination of papillary and follicular growth patterns. **(A)** Case 1: The tumor displayed noticeable nuclear enlargement, clearing, and irregularities in the cell membrane (H&E, x200). **(B)** Case 2: The tumor showed follicular epithelium hyperplasia with crowded nuclei (H&E, x200).

Both cases were diagnosed as the follicular variant of papillary thyroid carcinoma (FVPTC) at the time of diagnosis. They were characterized by the presence of multiple tumor nodules, invasive growth, and central lymph node metastases. Histopathological features of the two cases including: a combination of papillary and follicular growth patterns. The tumor is composed of small- to normal-sized follicles and has nuclear features of papillary thyroid carcinoma showing enlarged nuclei, irregular nuclear membranes, and chromatin clearing and glassy nuclei (**Figure 1**).

**Figure 2:**
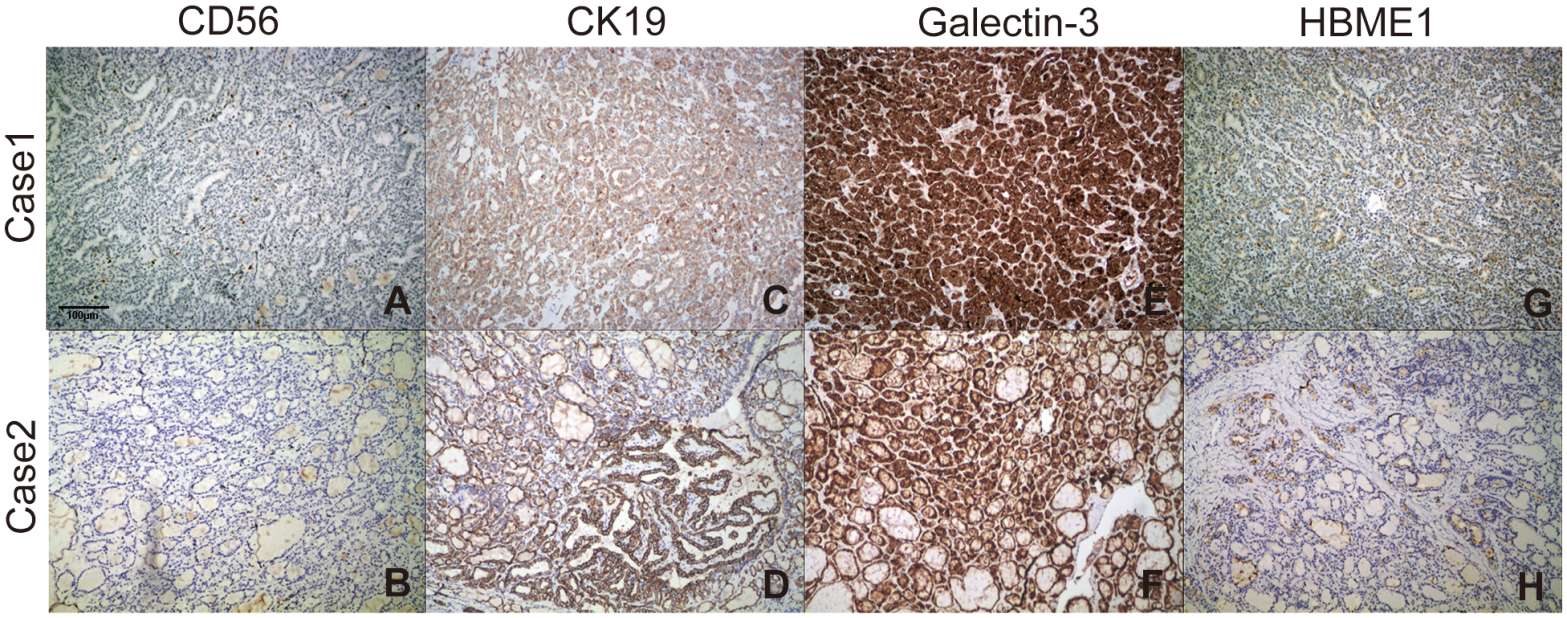
Staining profiles of CD56, CK19, Galectin-3, and HBME-1 in the two ETV6-NTRK3-rearranged FVPTC cases. **(A, B)** CD56 staining was consistently negative (100×). **(C, D)** CK19 staining was consistently positive (100×). **(E, F)** Galectin-3 exhibited strong diffuse staining in the cytoplasm and nucleus (100×). **(G, H)** HBME-1 staining showed sporadic positivity (100×).

**Figure 3:**
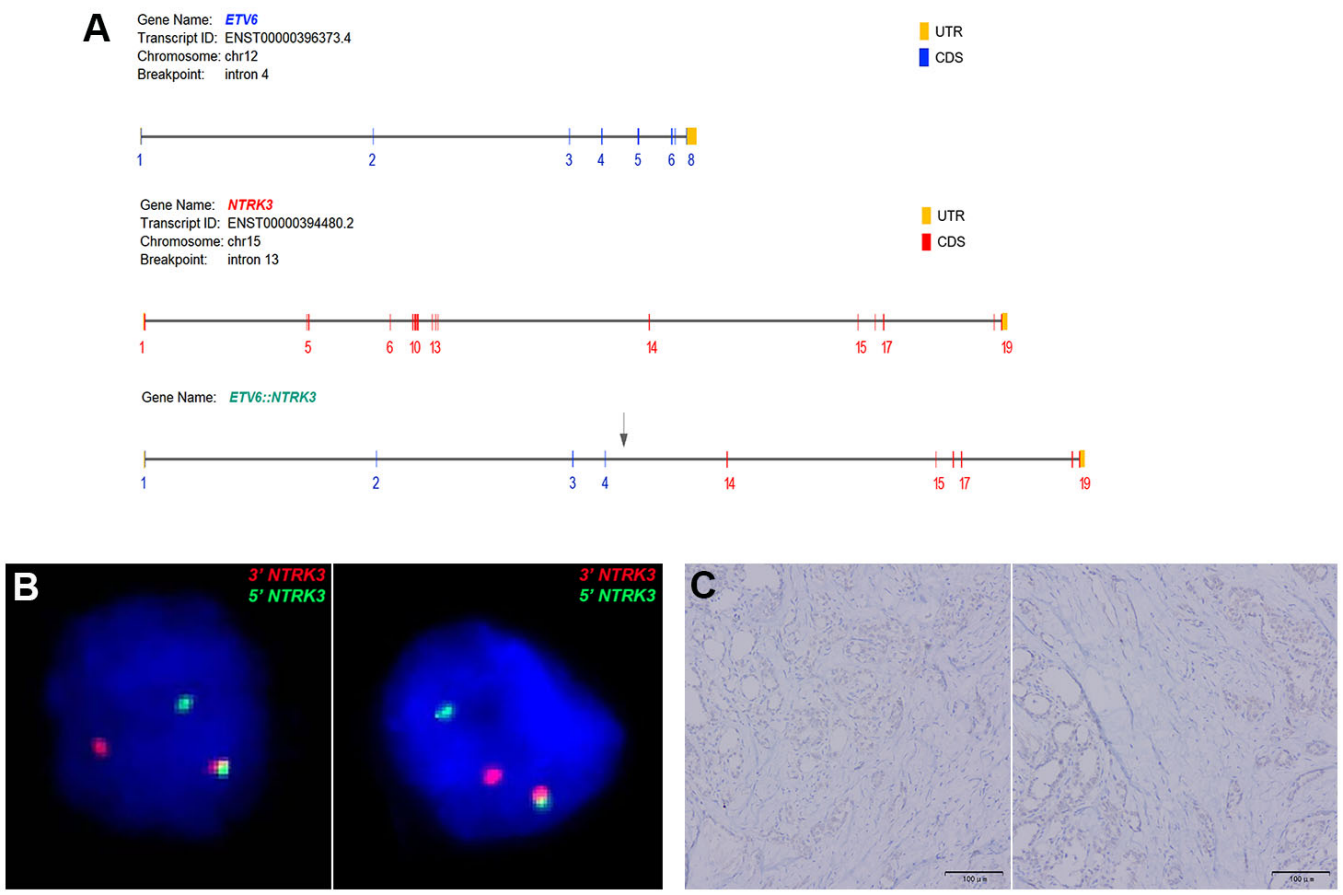
**(A)** Both cases of FVPTC demonstrated *ETV6::NTRK3* fusion with identical breakpoints, involving *ETV6* exon 4 and *NTRK3* exon 14. **(B)** FISH analysis using a dual-color break-apart *NTRK3* probe (green signal for 5’*NTRK3* and red signal for 3’*NTRK3*) confirmed *NTRK3* rearrangement. **(C)** Immunohistochemistry (IHC) using a Pan-TRK antibody revealed weak cytoplasmic and nuclear staining in *ETV6-NTRK3* rearranged thyroid cancer.

Immunohistochemical staining profiles showed CD56-, CK19+, Galectin-3+, HBME1+ (**Figure 2**). Both cases presented ETV6-NTRK3 rearrangements with the same fusion point (ETV6(E4)::NTRK3(E14)), the transcript ID of ETV6 and NTRK3 were ENST00000396373.4 and ENST00000394480.2 respectively. FISH analysis confirmed the rearrangement of NTRK3, with nuc ish (NTRK3×1~2) (3’NTRK3 sep 5’NTRK3×1) [34/50]. Additionally, Pan-TRK staining demonstrated positive results (**Figure 3**).

## 4 Discussion

Papillary carcinoma is the predominant pathological type of thyroid cancer in the Chinese population, accounting for over 90% (2). In addition to clinical staging, the behavior of papillary thyroid carcinoma (PTC) is also influenced by its pathological subtypes or genetic variants. In our study, we retrospectively reviewed medical records of thyroid cancer patients treated at our center from January 2021 to December 2022 and identified two of the patients showed NTRK rearrangement in their tumor tissues, specifically the *ETV6-NTRK3* rearrangement, both cases had the fusion of exon 4 of ETV6 to exon 14 of NTRK3, *ETV6*(E4)::*NTRK3*(E14), and were classified as follicular variant of papillary thyroid carcinoma (FVPTC). The correlation between these clinicopathological features and the molecular subtypes of *ETV6-NTRK3* rearrangements suggests that patients with similar clinicopathological features, especially those who are negative for *BRAF ^V600E^* mutation, should undergo further FISH or *ETV6-NTRK3* targeted sequencing.

In molecular pathological studies of thyroid cancer, the prevalence of *NTRK*-related thyroid cancer is relatively low. In a recent study, *NTRK1–3* rearrangements were identified in 13 (2.28%) out of 571 cases of thyroid cancer (18). Another study conducted on 496 adult thyroid tumors in TCGA reported a similar frequency, with NTRK1–3 rearrangements accounting for 2.34% (19). NTRK fusions are more frequently observed in specific populations, such as those with childhood papillary thyroid cancer (PTC) and PTC cases following the Chernobyl reactor accident, with reported frequencies ranging from 2% to 26% and 3% to 15% respectively (8, 20, 21). Ricarte-Filho et al. observed that NTRK3 fusion occurred in 25% (4/16) of FVPTC cases and in 5% (1/19) of classical PTC cases (22). Prasad et al. found that NTRK3 fusion was present in 100% (3/3) of FVPTC cases, 42.8% (3/7) of solid variant PTC cases, and 7.7% (1/13) of classic papillary PTC cases (8). These findings suggest a higher incidence of NTRK3 fusion in FVPTC compared to other subtypes of PTC.

The identified rearrangements in thyroid tumors so far include *EML4-NTRK3* (23), *ETV6-NTRK3* (8, 20, 22, 24), *IRF2BP2-NTRK1* (23), *TPR-NTRK1* (8, 22, 25), *TPM3-NTRK1* (21, 25), *TFG-NTRK1* (24, 25), *TRIM33-NTRK1* (24) and *SQSTM1-NTRK3* (23, 26). However, these studies have not established a clear association between the pathological types of thyroid cancer and molecular alterations. The two cases we discovered were both adult *ETV6-NTRK3* thyroid cancers, with no history of radiation exposure, and exhibited the pathological manifestations of follicular variant of papillary thyroid carcinoma (FVPTC). This specific pathological subtype was observed in both *ETV6-NTRK3* cases, suggesting a potential association between this pathological subtype and ETV6-NTRK3 thyroid cancer.

In our study, we applied immunohistochemical staining with CD56, HBME-1, CK19 and Galectin-3 for distinction of follicular variant of papillary carcinoma from follicular adenoma (FA) and follicular carcinoma. CD56 is a neural cell adhesion molecule (NCAM) that is normally expressed in thyroid follicular cells. Reduced expression of CD56 is associated with malignant tumors. Its expression is decreased or completely absent in papillary thyroid carcinoma, follicular carcinoma, and anaplastic carcinoma. CK19 expression is significantly higher in classical PTC and follicular variant PTC than in follicular thyroid carcinoma (FTC) and follicular adenoma, so it can be used to distinguish between thyroid papillary carcinoma and follicular adenoma. Galectin 3 plays a role in cell apoptosis regulation and cell movement, and is involved in the progression of thyroid cancer. HBME-1 expression in follicular variant PTC is significantly higher than in follicular adenoma or follicular carcinoma. The best combination for FVPTC cases was HBME-1, CD56, CK19 and Galectin 3, with a sensitivity reaching 91.1% (27).

FISH can detect large structural variations at the DNA level and is commonly used in clinical laboratories to detect oncogenic fusions in solid tumors. In theory, split probes have sufficient sensitivity and specificity for chromosomal abnormalities, but there are practical technical considerations in interpreting split FISH assays. Shorter split lengths are difficult to distinguish from split lengths in some normal cells, which can lead to false-negative results (28). Additionally, while positive results from split probes indicate the presence of structural variations involving the probe gene, it cannot be determined whether the abnormality leads to functional transcriptional fusion. The advantages of FISH include the small amount of material required, typically only a few unstained slides (usually one unstained slide per examination), and a turnaround time of only a few days (29).

In recent years, the application of immunohistochemistry (IHC) in the detection of NTRK rearrangements has been widely discussed. Studies have observed the clinical and pathological characteristics of *NTRK1/3* fusion PTC, examined the utility of pan-TRK IHC, and compared IHC with fluorescence *in situ* hybridization (FISH) and next-generation sequencing (NGS). It was found that pan-TRK IHC has a sensitivity of 58.3% and a specificity of 100% for *NTRK1/3* rearrangements in *BRAF^V600E^*-negative PTC. Pan-TRK IHC shows high specificity and moderate sensitivity for NTRK1/3 rearranged PTC, and should be interpreted with caution (30).

In conclusion, previous studies on *NTRK*-related thyroid cancers have demonstrated their molecular diversity, encompassing multiple subtypes, with papillary thyroid carcinomas (PTCs) being the most prevalent (31). Our study identified *ETV6-NTRK3* as the most common fusion type in PTC among the Chinese population. The pathological and immunohistochemical characteristics indicated that the follicular variant of papillary thyroid carcinoma (FVPTC) was the predominant subtype. Both cases examined exhibited a multi-nodular histological appearance and displayed an early tendency for extensive lymphovascular dissemination., These clinicopathological features suggest the possibility of *ETV6-NTRK3* rearranged thyroid carcinoma and emphasize the importance of conducting FISH or targeted RNA next-generation sequencing (NGS) for these patients. One of the limitations of our study is its retrospective nature, as our patients did not receive TRK inhibitors to provide information on treatment response. Previous studies have demonstrated that *ETV6-NTRK3* fusion tumors exhibit the highest sensitivity to entrectinib and larotrectinib, with minimal adverse effects (19). The incidence of grade 3 or 4 treatment-related adverse events, as defined by the Common Terminology Criteria for Adverse Events (CTCAE), is 15% (11, 15). Therefore, correlation studies between *ETV6-NTRK3* and clinicopathological characteristics are essential for identifying *ETV6-NTRK3* rearranged thyroid cancer based on clinicopathological manifestations, thereby providing greater clinical benefits to these patients.

## Data availability statement

The original contributions presented in the study are included in the article/supplementary material. Further inquiries can be directed to the corresponding author.

## Ethics statement

Written informed consent was obtained from the individual(s) for the publication of any potentially identifiable images or data included in this article.

## Author contributions

JinK: Resources, Writing – original draft, Writing – review & editing. HH: Data curation, Resources, Writing – review & editing. WZ: Writing – review & editing. PC: Methodology, Writing – review & editing. JHL: Methodology, Writing – review & editing. DS: Methodology, Writing – review & editing. SX: Conceptualization, Data curation, Writing – review & editing. JieK: Resources, Writing – review & editing. ZW: Resources, Writing – review & editing. JCL: Methodology, Resources, Writing – review & editing. YL: Writing – review & editing. MC: Writing – original draft, Writing – review & editing.
